# Protein methyltransferases and demethylases dictate CD8+ T-cell exclusion in squamous cell carcinoma of the head and neck

**DOI:** 10.18632/oncotarget.22627

**Published:** 2017-11-22

**Authors:** Theodore Vougiouklakis, Riyue Bao, Yusuke Nakamura, Vassiliki Saloura

**Affiliations:** ^1^ Department of Medicine, University of Chicago, Chicago, IL, USA; ^2^ Center for Research Bioinformatics, University of Chicago, Chicago, IL, USA; ^3^ Department of Pediatrics, University of Chicago, Chicago, IL, USA; ^4^ Department of Surgery, University of Chicago, Chicago, IL, USA; ^5^ Center for Cancer Research, National Cancer Institute, Chicago, IL, USA

**Keywords:** squamous cell carcinoma of head and neck, protein methyltransferases, immune markers

## Abstract

A subset of patients with recurrent/metastatic squamous cell carcinoma of the head and neck (SCCHN) benefit from pembrolizumab and nivolumab, but the majority of patients do not probably due to lack of activated cytotoxic CD8+ T-cells in their tumor tissues. Herein, we aim to investigate whether specific protein methyltransferases (PMTs) and demethylases (PDMTs) could play any roles in the CD8+ T-cell exclusion process in HPV-negative SCCHN. RNA sequencing data from the TCGA database were interrogated for HPV-negative SCCHN patients using a 10-gene chemokine signature that classifies SCCHN tissues into CD8+ T-cell inflamed and non-CD8+ T-cell inflamed phenotypes. Among 53 PMT/PDMT genes examined in the TCGA HPV-negative SCCHN database, expression levels of 15 PMT/PDMT genes were significantly negatively correlated with the chemokine signature score and CD8 mRNA expression levels. The expression level of each of these 15 PMT/PDMT genes showed significantly negative correlations with immune-active chemokines, as well as HLA class I and APM molecules. siRNA-mediated knockdown of a candidate PMT, SMYD3, led to upregulation of CXCL9, CXCL10, CXCL11 and TAP1 at mRNA and protein levels in HPV-negative SCCHN cell lines. These findings demonstrate that overexpression of some PMTs and PDMTs seems to be related with the non-CD8+ T-cell inflamed phenotype and may drive CD8+ T-cell exclusion in HPV-negative SCCHN. This study suggests that chromatin modifiers contribute to CD8+ T-cell exclusion and antigen presentation capacity of HPV-negative SCCHN, supporting that targeting of specific PMTs and/or PDMTs could enhance CD8+ T-cell infiltration and increase the proportion of patients that may benefit from immunotherapy.

## INTRODUCTION

Squamous cell carcinoma of the head and neck (SCCHN) is a frequent cancer type with approximately 50,000 patients diagnosed annually in the United States [1]. While HPV-positive, non-smoker patients with locoregionally advanced disease tend to have a relatively good prognosis [2], HPV-negative patients have an approximately 50% recurrence rate after treatment with standard chemoradiotherapy and/or surgery. Patients with recurrent/metastatic (R/M) SCCHN treated with standard platinum-based chemotherapy regimens have poor prognosis with a median overall survival of approximately 10 months [3]. Recently, pembrolizumab, a programmed-death-1 (PD-1) monoclonal antibody, was approved as a second-line therapy for R/M SCCHN after failure to platinum-based chemotherapy [4]. Although responses to pembrolizumab are as low as approximately 20% in PD-L1 positive tumors [4], 80% of the responders tend to have a durable response for more than 6 months, underlining the promise of immunotherapy as a novel therapeutic modality for SCCHN.

Dysregulation of the immune system plays a crucial role in the development and progression of SCCHN [5–7]. Immune escape in SCCHN occurs through various mechanisms, including (1) exclusion/restriction of cytotoxic CD8+ T-cell trafficking in the tumor microenvironment, (2) suppression of anti-tumor immune response through activation of immune checkpoint molecules and their receptors, or infiltration of the tumor tissue by immune suppressive cells such as T-regulatory cells (Tregs), myeloid-derived suppressor cells (MDSCs) and M2 macrophages, (3) loss of tumor-associated antigens through somatic mutations or decreased expression of HLA or the antigen presenting machinery (APM) components, and (4) lack of or decreased expression of immune active tumor-associated antigens. While multiple clinical trials are currently being conducted to reduce or overcome immune suppressive mechanisms in the tumor microenvironment, the molecular factors involved in CD8+ T-cell exclusion and ineffective tumor-associated antigen presentation in SCCHN are still not well understood.

Epigenetic regulation mediated by histone modifications has emerged as an attractive therapeutic avenue for anticancer therapy. The Cancer Genome Atlas (TCGA) recently revealed a plethora of genetic alterations in chromatin modifiers in multiple cancer types, including SCCHN. Of these chromatin modifiers, protein methyltransferases (PMTs) and demethylases (PDMTs) have attracted a major interest as novel targets for drug development. A significant body of preclinical evidence supports their oncogenic function both as epigenetic regulators through histone methylation, but also through non-histone protein methylation [8, 9]. In addition to these functions, recent evidence supports that PMTs may be involved in the regulation of immune-cell trafficking to the tumor microenvironment. More specifically, a recent study showed that the PMT enhancer of zeste homologue 2 (EZH2) and DNA-methyltransferase 1 (DNMT-1) transcriptionally repress Th1-type chemokines, CXCL9 and CXCL10, and that inhibition of both EZH2 and DNMT-1 increased CD8+ T-cell trafficking, reduced tumor growth and improved the efficacy of PD-L1 checkpoint blockade in a mouse ovarian cancer model [10]. Similarly, Nagarsheth et al. [11] found that EZH2 transcriptionally represses the expression and production of CXCL9 and CXCL10 through H3K27 tri-methylation in colon cancer cells, and that overexpression of EZH2 was associated with decreased CD8+ and CD4+ T-cell tumor infiltration as well as poor prognosis in colon cancer patients.

HLA class I and APM molecules have also been reported to be transcriptionally regulated through promoter DNA-methylation [12]. Transcriptional downregulation was also described as a mechanism of immune evasion of cancer cells [13]. Mora-Garcia et al. [14] reported that cervical cancer cells treated with a demethylating agent and a histone deacetylase inhibitor showed decreased promoter DNA-methylation of HLA class I genes and enhanced the activity of antigen-specific cytotoxic T-lymphocytes. However, no studies have systematically assessed the effect of PMTs/PDMTs on the transcriptional control of HLA class I cluster or the APM genes.

In this study, we attempted to investigate the possibility that PMTs or PDMTs might regulate effector T-cell trafficking as well as the antigen presentation machinery in HPV-negative SCCHN. Specifically, we hypothesized that overexpression of specific PMTs or PDMTs by cancer cells may silence the transcription and expression of effector T-cell chemokines or HLA class I and APM components, leading to decreased effector T-cell trafficking and/or defective antigen presentation in SCCHN. Using a recently described chemokine signature which stratifies CD8+ T-cell inflamed locoregionally advanced SCCHN tumors from non-T-cell inflamed tumors [15, 16], we have interrogated expression levels of PMTs and PDMTs across two large mRNA microarray HPV-negative SCCHN databases in order to identify chromatin modifiers that are positively associated with the non-T-cell inflamed SCCHN phenotype and could thus be biological culprits for CD8+ T-cell exclusion or decreased antigen presentation in HPV-negative SCCHN.

## RESULTS

### Correlation of expression levels of PMTs and PDMTs with immune cell signatures and the non-T-cell inflamed phenotype of HPV-negative SCCHN

**Figure 1:**
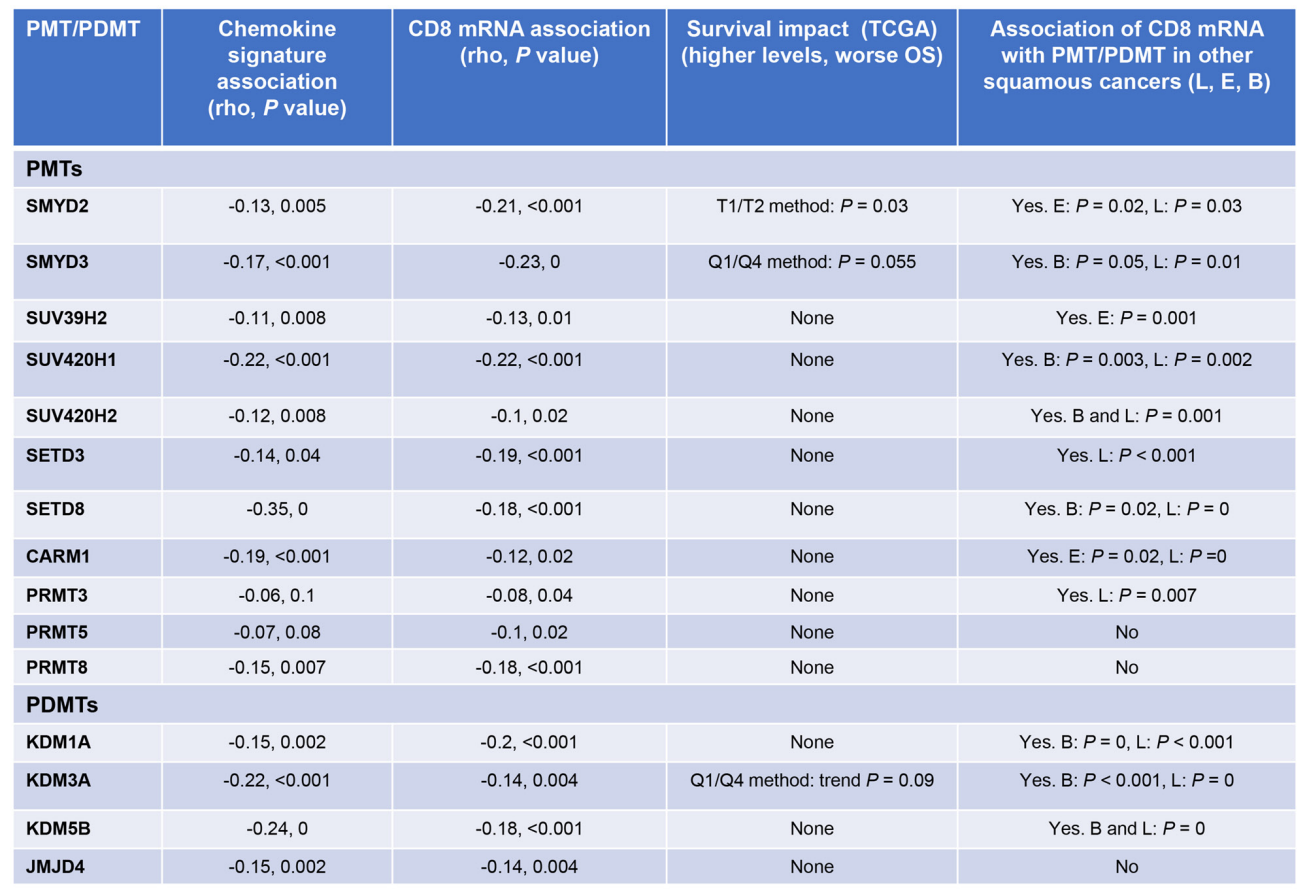
PMTs/PDMTs that are negatively correlated with the mean chemokine signature scores and CD8 mRNA Pearson's correlation coefficients were calculated, and correlations were considered significant if *P* < 0.05. Overall survival in SCCHN and associations of PMT/PDMT with CD8 mRNA in lung squamous, esophageal squamous and bladder carcinoma are also shown. Correlations were performed using the TCGA mRNA database. Overall survival analysis was performed with the Kaplan-Meier method (median, quartile or percentile) using the TCGA mRNA database for SCCHN, lung, esophageal squamous and bladder carcinoma. PMT: protein methyltransferase, PDMT: protein demethylase, OS: overall survival, L: lung squamous, E: esophageal squamous, B: bladder.

Of the 53 PMT/PDMT genes examined, expression levels of 11 PMT and 4 PDMT genes were negatively correlated with the mean chemokine signature score (*P* < 0.05), which was defined as the arithmetic mean of normalized & log2-transformed expression of the chemokine genes (CCL2, CCL3, CCL4, CCL5, CCL19, CCL21, CXCL9, CXCL10, CXCL11, CXCL13), calculated by (gene 1 expression + gene 2 expression +… gene 10 expression)/10. All 15 genes were also confirmed to correlate negatively with CD8 mRNA levels (Figure 1). 12 of these PMT/PDMT genes were further confirmed to correlate negatively with CD8 mRNA expression in lung squamous, esophageal squamous and bladder cancer. Furthermore, higher expression levels of three genes, SMYD2, SMYD3 and KDM3A, were associated or showed a trend for worse overall survival in HPV-negative SCCHN patients, though the chemokine signature or CD8 mRNA levels were not associated with overall survival.

**Figure 2:**
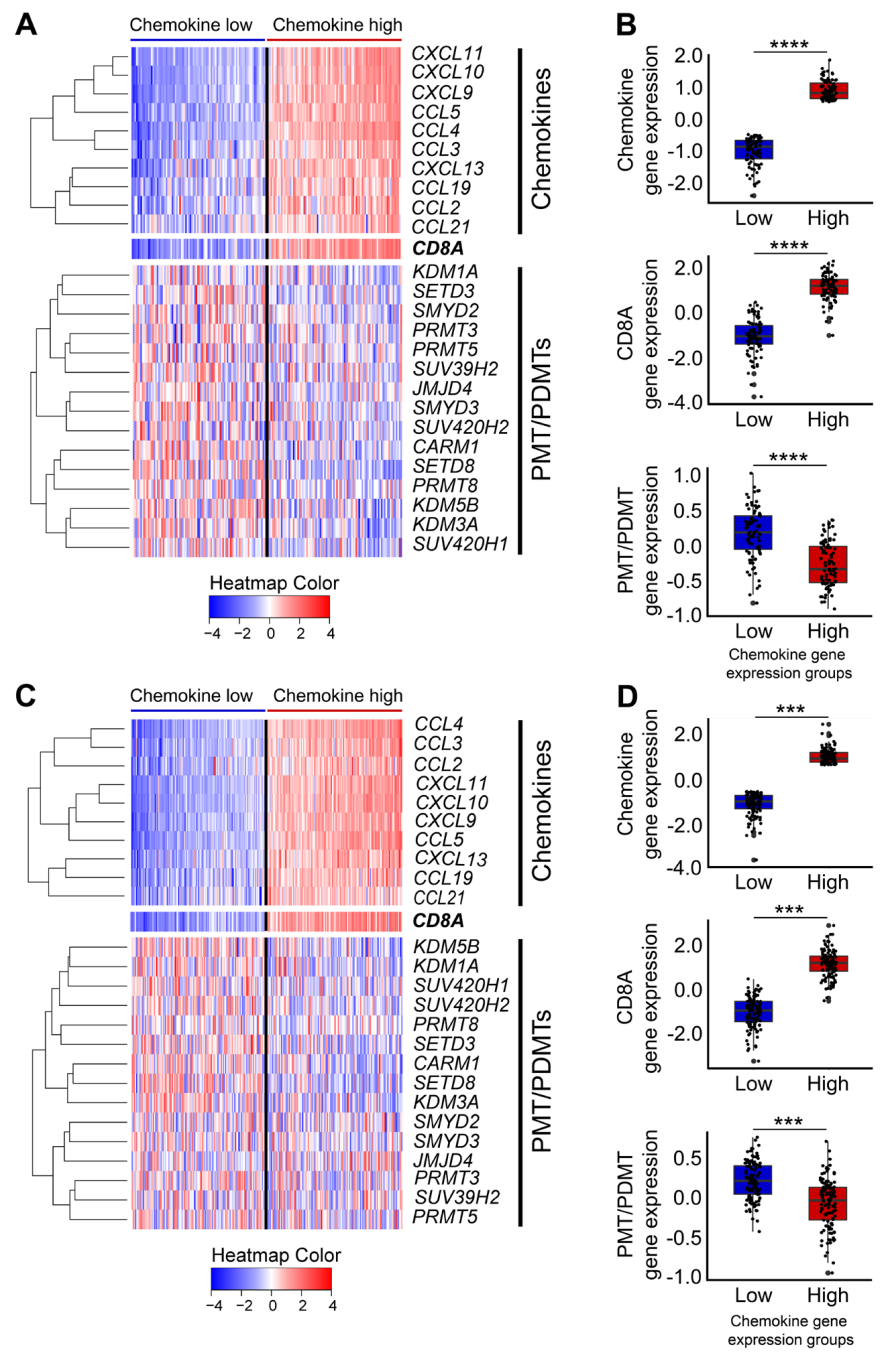
Heatmap of 15 candidate PMTs/PDMTs with the chemokine signature in the TCGA database for HPV-negative SCCHN and lung squamous cell carcinoma Heatmaps were generated using the normalized and log2-transformed RNA-seq gene expression data. **(A-B)** HPV-negative SCCHN. (A) Panel A, top: Expression heatmap of the 10 chemokine genes (left to right: samples were sorted by median chemokine gene expression, lower to higher). Panel A, middle: Expression heatmap of CD8A (same sample order as the chemokine gene heatmap). Panel A, bottom: Expression heatmap of the 15 PMT/PDMT genes (same sample order as the chemokine gene heatmap). (B) Box plot showing the expression of chemokines, CD8A and PMT/PDMT genes in the T-cell inflamed and non-T-cell inflamed groups, indicated by low and high chemokine gene expression, respectively, which highly correlates with CD8A+ gene expression. Top to bottom: Box plot showing the median chemokine gene expression, CD8A gene expression, and median PMT/PDMT gene expression in the two tumor groups (^****^ *P* < 0.0001). **(C-D)** Lung squamous cell carcinoma. As per panels (A-B). (^***^ *P* < 0.001).

To further examine differences in expression levels of these 15 PMT/PDMT genes between the non-CD8+ T-cell inflamed and the T cell-inflamed tumors, we classified the expression levels of chemokine genes in the HPV-negative SCCHN patients into four groups (Q1 to Q4, from low to high). For the current analysis, we compared two groups, the lowest one-fourth (Q1, chemokine low) and the highest one-fourth (Q4, chemokine high) and found that expression levels of these 15 genes were significantly higher in the chemokine low group, representative of the non-CD8+ T-cell inflamed phenotype, than in the chemokine high group (Figure 2A, 2B; Supplementary Figure 1). This difference was reproducible in tumors in the Chicago Genomics Cohort (CHGC) (Supplementary Figure 2) and was also observed in the lung squamous (Figure 2C, 2D; Supplementary Figure 3) and bladder cancers in the TCGA database (Supplementary Figure 4), suggesting that these 15 PMT/PDMT genes might play some roles in the CD8+ T-cell exclusion process in HPV-negative SCCHN.

### Expression of specific PMTs and PDMTs are negatively correlated with the expression levels of chemokines, HLA class I and/or APM molecules in HPV-negative SCCHN

**Figure 3:**
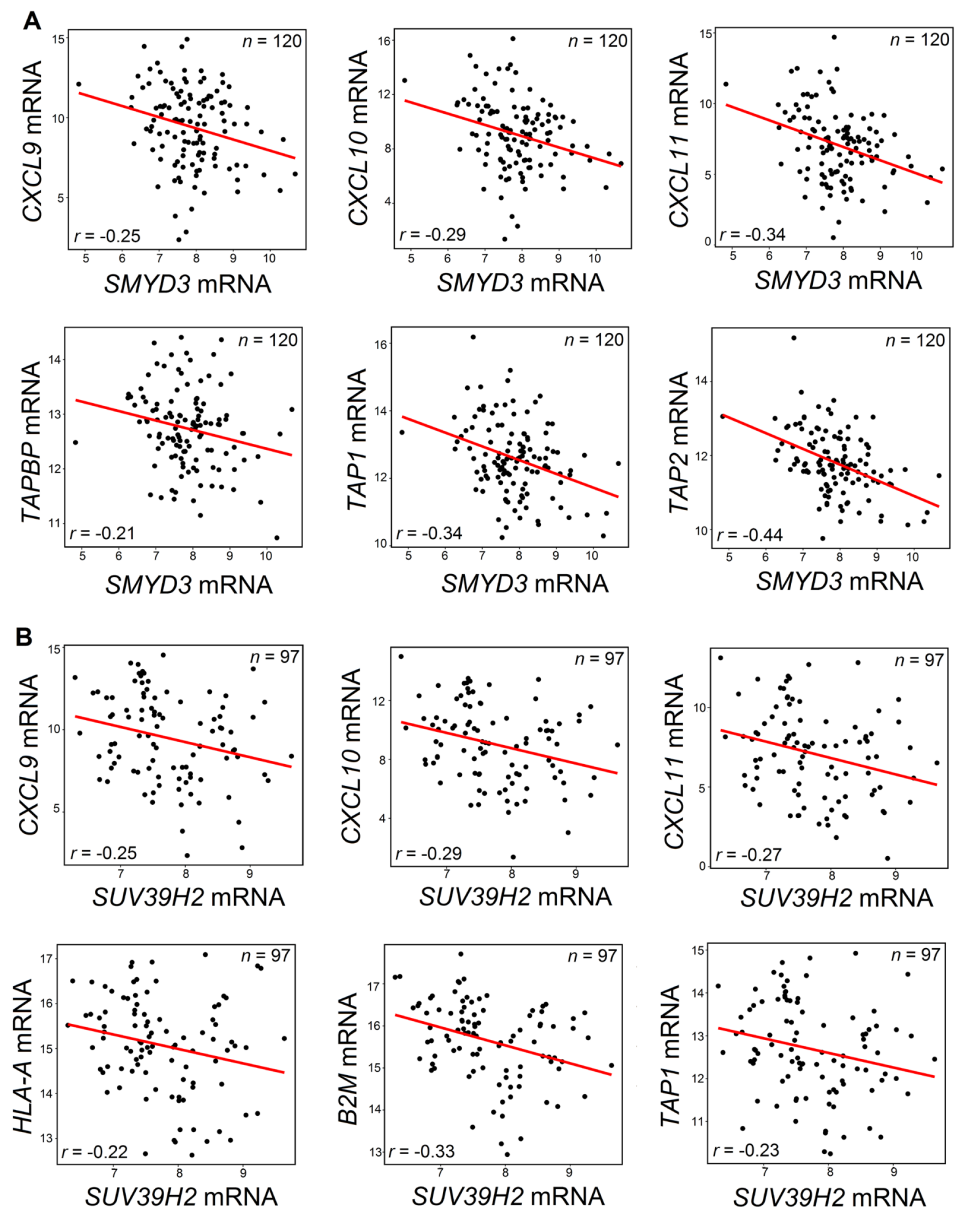
Negative correlations between SMYD3 (A) or SUV39H2 (B) and chemokines or APM molecules **(A)** SMYD3 mRNA was negatively correlated with mRNA levels of CXCL9, CXCL10 and CXCL11 chemokines, and with mRNA levels of TAPBP, TAP1 and TAP2 using the TCGA database for SCCHN. **(B)** SUV39H2 mRNA was negatively correlated with mRNA levels of CXCL9, CXCL10 and CXCL11 chemokines, and with mRNA levels of HLA-A, B2M and TAP1 using the TCGA database for SCCHN. Pearson's correlation coefficients *r* were calculated and significance was defined as *P* < 0.05. All shown results were statistically significant.

We hypothesized that PMT/PDMT genes overexpressed in SCCHN cancer cells may be involved in CD8+ T-cell exclusion from SCCHN tissues through transcriptional silencing of chemokines, or by reducing antigen presentation through transcriptional silencing of the HLA class I and/or APM molecules. Hence, we examined whether the 15 PMT/PDMT genes, which revealed negative correlations with CD8 expression, would also be negatively correlated with chemokines, HLA class I or APM molecules. In our analysis, we included PMTs and PDMTs that are known to induce not only repressive histone marks but also activating marks, as it is possible that activating marks may induce the expression of miRNAs that could silence specific chemokines, HLA class I or APM molecules. As shown in Table 1, expression levels of most of 15 PMTs and PDMTs genes seemed to correlate negatively with expression levels of multiple chemokines and/or HLA class I and APM molecules. Figure 3 shows examples of negative correlations between expression levels of protein methyltransferases SMYD3 (Figure 3A), SUV39H2 (Figure 3B), and chemokines, HLA class I and APM molecules.

**Table 1:** PMT/PDMTs that significantly negatively correlate (*P* < 0.05) with chemokines, HLA-class I or APM molecules

PMT/PDMTs	Chemokines	HLA class I and APM molecules
**PMTs**		
**SMYD2**	CCL2	
**SMYD3**	CXCL9, CXCL10, CXCL11, CCL5, CCL21	TAP1, TAP2, TAPBP, IRF1, HLA-A/B/C, B2M
**SUV39H2**	CCL4, CXCL9, CXCL10, CXCL11	B2M, HLA-A/B/C, IRF1, TAP1, TAP2
**SUV420H1**	CCL4, CCL5, CCL21, CXCL11	B2M, HLA-A/B/C, IRF1, TAP1, TAPBP
**SUV420H2**	CCL2, CCL19	
**PRDM9**	CCL2, CCL5, CCL19, CXCL9, CXCL13	CANX, TAP1, TAPBP
**SETD3**	CCL2, CCL19, CCL21, CXCL13	
**SETD8**	CCL2	B2M, CALR, CANX, HLA-A/B/C, TAP1, TAP2
**CARM1**		B2M, TAPBP, TAP1, TAP2, CANX, HLA-A/B/C, IRF1
**PRMT3**		TAPBP, CALR
**PRMT5**	CCL2, CXCL11	TAPBP
**PRMT8**	CCL19	TAPBP
**PDMTs**		
**KDM1A**	CCL2, CCL19	
**KDM3A**	CCL3, CCL5, CXCL11	HLA-A/B/C, TAP1, TAP2, CALR, B2M
**KDM5B**	CCL2, CCL3, CCL4, CCL21, CXCL13	
**JMJD4**		CANX

Abbreviations: PMTs/PDMTs: protein methyltransferases/protein demethylases, HPV: human papilloma virus, SCCHN: squamous cell carcinoma of the head and neck, APM: antigen presenting machinery, CHGC: Chicago Genomics Cohort, SCC: squamous cell carcinoma, CCLE: Cancer Cell Line Encyclopedia.

Considering the possibility that PMT/PDMT genes may be expressed not only in cancer cells, but also in tumor stroma cells, including antigen presenting cells, we further validated the negative correlation results using the RNA sequencing database of the Cancer Cell Line Encyclopedia (CCLE) for squamous cell carcinoma, lung squamous and esophageal squamous cell lines (*n* = 184). This additional analysis confirmed most of negative correlations shown in Table 1 (Supplementary Table 4); for example, negative correlation of expression levels of SMYD3 with those of CXCL10 (Pearson's correlation coefficient *r* = -0.25, *P* < 0.001 in the TCGA SCCHN database) was confirmed in the CCLE database with *r* = -0.31 and *P* = 0.004. Some correlations were not confirmed in CCLE database and need further verification, though a possible reason could be that stroma cells may be predominantly driving these relationships.

### SMYD3 knockdown induces transcriptional upregulation of CD8+ T-cell attracting chemokines and APM components in SCCHN cells

**Figure 4:**
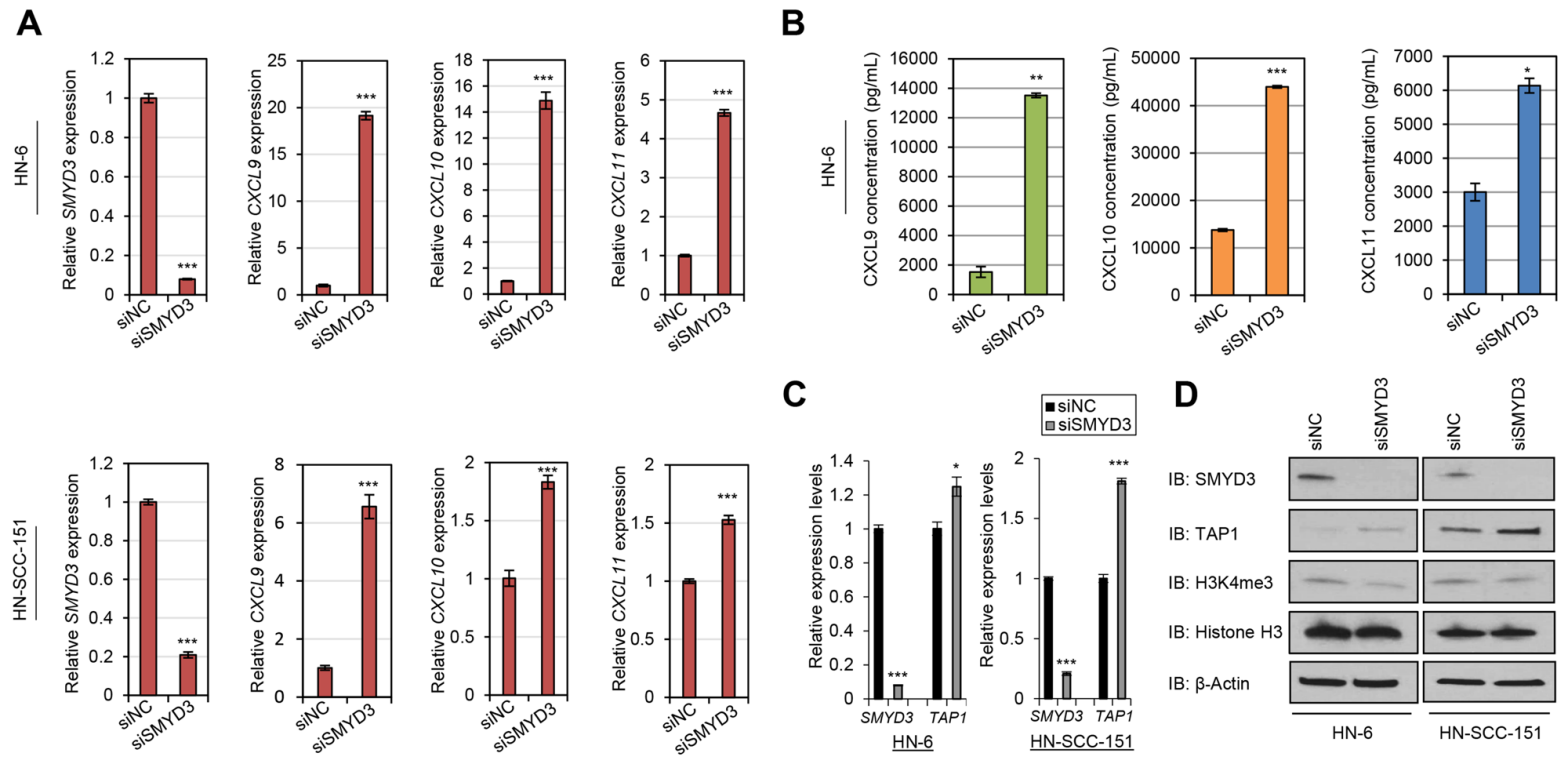
*SMYD3* knockdown induces the expression of CD8+ T-cell attracting chemokines and antigen processing machinery molecules in SCCHN cells **(A-B)** Induction of *CXCL9*, *CXCL10* and *CXCL11* mRNA transcripts and protein levels upon *SMYD3* knockdown in SCCHN cell lines. HN-6 and HN-SCC-151 cells were transfected with a control siRNA (siNC) and a SMYD3-specifc siRNA (siSMYD3) for a period of 72 hours. 24 hours prior to collection, cells were exposed to 1000U/mL of IFN-β. (A) *SMYD3* knockdown resulted in significant upregulation of *CXCL9*, *CXCL10* and *CXCL11* mRNA levels in HN-6 and HN-SCC-151 cells assessed by real-time PCR. (^***^P < 0.001). mRNA levels were normalized by *GAPDH*. (B) Effect of *SMYD3* knockdown on IFN-β-mediated release of CXCL9, CXCL10 and CXCL11 chemokines in SCCHN cells. Supernatants collected from HN-6 cells were further assayed for CXCL9, CXCL10 and CXCL11 protein levels by ELISA. Cells treated with siSMYD3 showed increased protein levels of CXCL9, CXCL10 and CXCL11 production compared to siNC treated cells. (^*^P < 0.05; ^**^P < 0.01; ^***^P < 0.001). **(C-D)** Induction of *TAP1* mRNA transcripts and protein levels upon *SMYD3* knockdown in SCCHN cell lines. HN-6 and HN-SCC-151 cells were transfected with a control siRNA (siNC) and a SMYD3-specifc siRNA (siSMYD3) for a period of 72 hours. 24 hours prior to collection, cells were exposed to 1000U/mL of IFN-β. (C) Real-time PCR demonstrates upregulation of *TAP1* after *SMYD3* knockdown (same samples as in (A)) (^*^P < 0.05; ^***^P < 0.001). mRNA levels were normalized by *GAPDH*. (D) Western blotting of nuclear (SMYD3, H3K4me3) and cytoplasmic (TAP1) extracts shows an increase in TAP1 protein levels and attenuation of H3K4me3 levels upon *SMYD3* knockdown.

Among the 15 PMT/PDMT genes, SMYD3 revealed significantly negative correlations with multiple chemokines, HLA class I and APM molecules (Table 1). Additionally, SMYD3 has been described as an oncogenic methyltransferase since 2004 [20], and many studies have further supported its significance in human tumorigenesis in multiple cancer types [21–24]. Based on this preclinical evidence, SMYD3 has been under investigation as an anticancer target for the identification of specific drug compounds, and SMYD3 inhibitors that suppress the growth of SMYD3-overexpressing cancer cells were recently reported in the literature [25, 26]. Hence, we focused on SMYD3 for the further biological analysis. First, we transfected two HPV-negative SCCHN cell lines, HN-6 and HN-SCC-151, with a SMYD3-specific siRNA (siSMYD3) and a control siRNA (siNC) for 72 hours. 24 hours prior to collection, cells were exposed to 1000U/mL of IFN-β. We collected cell pellets, extracted RNA, and performed real-time PCR for specific chemokines, HLA class I molecules and APM components that were found to be negatively correlated with SMYD3, as discussed above. siRNA-mediated knockdown of *SMYD3* led to upregulation of the mRNA transcripts of CD8+ T-cell attracting chemokines CXCL9, CXCL10 and CXCL11 in both HN-6 and HN-SCC-151 cell lines (Figure 4A). We subsequently examined the protein levels of these chemokines by ELISA assays using supernatants from the previously transfected HN-6 cells. *SMYD3* knockdown resulted in a significant increase of CXCL9, CXCL10 and CXCL11 protein levels, compared with the siNC group (Figure 4B). Under similar experimental conditions, we also assessed the effect of siRNA-mediated knockdown of *SMYD3* on the expression levels of HLA class I and APM molecules. *SMYD3* knockdown downregulated tri-methylated H3K4 (H3K4me3) and resulted in the increase of *TAP1* transcript and protein levels in HN-6 and HN-SCC-151 cell lines (Figure 4C, 4D). Taking into consideration that H3K4 tri-methylation is an activation mark, these findings suggest that the transcription of CXCL9, CXCL10, CXCL11 and TAP1 may be regulated by SMYD3 indirectly through miRNAs or the expression of chromatin modifiers that induce suppressive marks.

## DISCUSSION

Immune checkpoint blockade has gained accelerated approval in the treatment of multiple cancer types, such as melanoma, lung, bladder, renal cell carcinoma and SCCHN. Although responders tend to attain significantly prolonged survival, this percentage is limited with a range of approximately 10-25% of cancer patients. One of the reasons for this low response rate is that multiple layers of positive and negative immune feedback systems, including multiple immune checkpoints and immune suppressive cells, are engaged within the tumor microenvironment as mechanisms of de novo or adaptive tumor immune resistance. For this reason, multiple clinical trials are currently underway investigating different combination regimens of immune checkpoint blockade. While in some tumors this may be a predominant mechanism of immune evasion, other tumors may utilize mechanisms that lead to effector CD8+ T-cell exclusion or decreased capacity for antigen presentation. Harlin et al. [15] previously demonstrated that the production of a subset of chemokines by melanoma cells was essential for the induction of T-cell infiltration in metastatic melanoma lesions and concordantly, the absence of expression of these chemokines was associated with a non-T-cell inflamed phenotype. Understanding the molecular mechanisms that coordinate and induce the expression of these chemokines from cancer cells is thus instrumental to improve the efficacy of immune checkpoint blockade and increase the percentage of patients that could benefit from immunotherapy.

Recent work has attempted to shed light on potential mechanisms that could drive T-cell exclusion from tumors [27]. Studies in melanoma models have underlined the pivotal role of innate immunity in the generation of a spontaneous antitumor T-cell response by showing that production of type I IFN-β by host dendritic cells in the tumor microenvironment was necessary for the priming of tumor-specific T-cells [28]. The same group also found that the adapter stimulator of interferon genes complex (STING) is directly activated by tumor-derived cytosolic DNA present within dendritic cells and triggers the activation of IFN-β production, thus enabling innate immune recognition of immunogenic tumors and subsequent effector T-cell infiltration [29]. Another focus has also been the interrogation of specific genomic alterations that could contribute to T-cell exclusion. Activating STAT3 mutations have been shown to block chemokine expression in melanoma cells and cause T-cell exclusion [30]. Furthermore, activation of the β-catenin pathway is sufficient to exclude T-cells from mouse melanoma tumors [31]. With further interrogation into the specific genomic landscape of each tumor type, it is possible that different mechanisms may be responsible for T-cell exclusion in different cancer types.

Recently, epigenetic modifiers such as azacytidine (AZA) (DNA methyltransferase inhibitor) and histone deacetylase (HDAC) inhibitors, have attracted attention as potential modulators of antitumor immune responses. This idea has its roots in experiments performed over a decade ago, when Karfp et al. [32] showed that treatment of colon cancer cell lines with AZA induced the expression of HLA class I, tumor-associated antigen and interferon pathway genes. Furthermore, Kim et al. [33] recently showed that the combination of the HDAC inhibitor entinostat and AZA, with anti-PD-1 and anti-CTLA4 achieved complete regression of tumors in mouse models of colorectal and breast cancer, and this was attributed to a significant decrease in the myeloid derived suppressor cells in the tumors. In another study, Chiappianelli et al. [34] showed that DNA methyltransferase inhibitors upregulate the expression of endogenous retroviral genes, and that this triggers a type I IFN response and potentiates the antitumor effects of anti-CTLA4 treatment in a murine melanoma model. With mounting preclinical evidence, a number of clinical trials have been launched to investigate the combination of AZA or HDAC inhibition with immune checkpoint inhibitors. That being said, there are concerns that AZA and HDAC inhibitors have diverse effects and off-target functions which are still elusive in cancer and immune cells.

PMTs and PDMTs, a large class of epigenetic modulators, are frequently genetically altered in a wide variety of cancer types and function through direct methylation of histone and non-histone proteins. Other than the work performed by the Zou group [10, 11] showing that EZH2 produced by colon cancer cells mediates transcriptional silencing of Th1-type CXCL9 and CXCL10 chemokines, and causes subsequent CD8+ T-cell exclusion, no other studies have systematically explored the potential function of PMTs and PDMTs as one of the key factors of CD8+ T-cell exclusion or antigenicity in solid tumors. Using the chemokine signature described previously [19], we recently showed that in HPV-negative SCCHN, 79% of patients exhibit a non-CD8+ T-cell inflamed phenotype [16], indicating that T-cell exclusion and/or decreased antigenicity may be primary determinants of decreased efficacy of immune checkpoint blockade in patients with HPV-negative SCCHN.

We hypothesized that specific PMT/PDMTs produced by cancer cells may silence the transcription of effector T-cell chemokines or HLA class I and APM components, leading to decreased effector T-cell trafficking and/or reduced antigen presentation in SCCHN. The goal of this study was to explore and provide proof of concept for further investigation into mechanisms of T-cell exclusion and/or modulation of cancer antigen presentation that could be mediated through specific PMT/PDMT enzymes. We showed that 11 PMTs and 4 PDMTs appear to be negatively correlated with the CD8+ T-cell inflamed SCCHN phenotype. Furthermore, we showed that these PMT/PDMTs also correlated negatively with various CD8+ T-cell attracting chemokines, HLA class I and/or APM components. We confirmed these negative correlations using the CCLE database of head and neck, lung, and esophageal squamous cancer cell lines, suggesting that PMT/PDMTs produced by cancer cells, and not necessarily only by immune stroma cells, may drive CD8+ T-cell exclusion and decrease the antigen presentation. We further validated and functionally analyzed the negative correlation of one of these PMTs, SMYD3, with chemokines and APM components, by showing that siRNA-mediated knockdown of *SMYD3* led to upregulation of the transcript and protein levels of CXCL9, CXCL10, CXCL11 and TAP1 in two HPV-negative SCCHN cell lines. Given that SMYD3 is known to write an activation mark, that is H3K4 tri-methylation, which directly induces the transcription of target genes, our hypothesis is that SMYD3 indirectly regulates the expression of the chemokines and TAP1 through miRNAs or chromatin modifiers that induce suppressive marks. Our lab is pursuing further investigation of mechanisms pertaining to this hypothesis.

This is the first study to systematically examine the potential contribution of PMT/PDMT chromatin modifiers in CD8+ T-cell exclusion and the antigen presentation capacity of HPV-negative SCCHN and to lay the scientific rationale to pursue further investigation into relevant mechanisms. A significant limitation of this study is its exploratory, correlative nature and the need for additional validation of all mRNA associations at the protein level in SCCHN tissue samples. In this context, our lab is currently investigating the specific mechanisms through which SMYD3 regulates the expression of CD8+ T-cell attracting chemokines and APM components in SCCHN cells. Furthermore, it would be of paramount importance to determine whether PMTs affect tumor T-cell infiltration or tumor immunogenicity through direct methylation of substrates involved in these processes [35]. As some PMT/PDMT inhibitors are expected to enter early phase clinical trials, investigating the potential effect of these enzymes as modifiers of tumor immunogenicity could contribute significantly to increase the percentage of cancer patients that benefit from immunotherapy.

## MATERIALS AND METHODS

### Patient cohorts

The TCGA RNA-seq gene expression data were obtained from Broad Genome Data Analysis Center (GDAC; release date November 1, 2015, level 4) and were used to assess mRNA expression correlations for early stage SCCHN [17] (*n* = 503; HPV-negative = 364), lung squamous cell (*n* = 488), esophageal squamous cell (*n* = 170) and bladder carcinomas (*n* = 344). RNA-seq raw read counts were summarized at gene level using the expectation maximization (RSEM) method, followed by upper quartile-normalization and log2 transformation. Given the low number of HPV-positive patients, we decided to concentrate our analysis on HPV-negative patients. The Chicago Genomics Cohort (CHGC) previously described by Keck et al. [18] was used as a validation cohort (HPV-negative, *n* = 73).

### Bioinformatics analysis

We sought to investigate correlations between 53 PMT/PDMT genes and CD8+ T-cell attracting chemokines as well as APM components (Supplementary Table 1), in order to identify candidate PMT/PDMT genes that could be mechanistically responsible for the exclusion of CD8+ T-cells in SCCHN through transcriptional downregulation of chemokines or APM components. To this purpose, RNA sequencing expression data were initially used from the TCGA database of HPV-negative SCCHN tissue samples (*n* = 364). The candidate PMT/PDMT genes were interrogated across SCCHN tissue samples from the TCGA database using components of a chemokine signature (CCL2, 3, 4, 5, 19, 21, CXCL9, CXCL10, CXCL11, CXCL13) which was previously reported to classify different cancer types, including SCCHN, to CD8+ T-cell inflamed versus non-T-cell inflamed phenotypes [19]. In the same context, Harlin et al. [15] had previously shown that metastatic melanoma cells expressing components of this chemokine signature induced migration of CD8+ T-cells within melanoma metastasis, whereas lack of expression of this subset of chemokines correlated with lack of CD8+ T-cell infiltration. Using this chemokine signature, we previously showed that locoregionally advanced SCCHN tumors could be stratified to a CD8+ T-cell inflamed versus a non-CD8+ T-cell inflamed phenotype [16]. Correlations between the mRNA expression levels of the candidate PMT/PDMT genes and the mean chemokine signature scores were pursued. The PMT/PDMTs were further sorted out based on the hypothesis that if these genes induce CD8+ T-cell exclusion, they should exhibit a significant negative correlation (*P* < 0.05) with CD8 mRNA. Additionally, we hypothesized that PMT/PDMTs that induce CD8+ T-cell exclusion would be associated with worse overall survival. The survival analysis was performed using the Kaplan-Meier method. Survival difference was compared between groups and statistical significance was calculated using log-rank test. The selected PMT/PDMT genes were further validated by exploring the relationship of each PMT/PDMT with these signatures and CD8 mRNA in the CHGC database, as well as the TCGA database of lung squamous, esophageal squamous and bladder cancer, which have similar genetic background with SCCHN.

Subsequently, the mRNA expression levels of the PMT/PDMT genes that showed significant negative correlation with the chemokine signature and CD8 mRNA were correlated with the mRNA expression levels of the individual chemokine signature components, as well as the HLA class I (A, B, C) and the APM molecules β2-microglobulin, transporter associated with antigen protein 1 and 2 (TAP1, TAP2), low molecular weight protein 2 and 7 (LMP2/7), calretinin (CALR), calnexin (CANX), and tapasin (TAPBP). Any genes with significant negative correlations (*P* < 0.05) were selected for further exploration.

Given that the mRNA from these databases is derived not only from tumor cells but also immune stromal cells, and our hypothesis is that PMT/PDMTs driving CD8+ T-cell exclusion are produced by cancer cells, we sought to further validate negative correlations between the selected PMT/PDMT genes and chemokines, HLA class I and/or APM components in the Cancer Cell Line Encyclopedia mRNA database using SCCHN, lung squamous and esophageal squamous cancer cell lines with available mRNA expression data. Table 2 shows a flow chart of the methodology used to sort out promising PMT/PDMTs for further preclinical investigation.

**Table 2:** Flow chart of methodology to sort out PMT/PDMTs that could be responsible for CD8+ T-cell exclusion in HPV-negative SCCHN

Run associations of 53 candidate PMTs/PDMTs using the TCGA mRNA database for HPV-negative SCCHN with the mean chemokine signature score
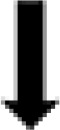
Sort out candidate PMT/PDMTs as follows: 1) Genes that show a significant negative correlation with the mean chemokine signature score **AND** 2) Genes that show a significant negative correlation with CD8 mRNA (*P* < 0.05)
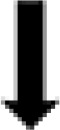
Genes that show a statistically significant negative correlation (*P* < 0.05) with any of the components of the chemokine signature, and/or HLA-I (A, B, C) and APM components
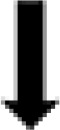
Further validate selected PMT/PDMT genes with the following filters: (a) CHGC (b) Lung SCC, esophageal SCC, bladder cancer TCGA databases (c) CCLE database (negative correlations with chemokines, HLA class I, APM components
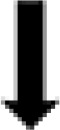 Initial preclinical validation Using cell culture systems, knockdown candidate PMT/PDMT(s) in human SCCHN cells and assess expression of chemokines, HLA-I and/or APM components at mRNA and protein level

### Cell culture

Squamous cell carcinoma cell lines HN-6 and HN-SCC-151 were derived from patients with locoregionally advanced SCCHN and were kindly provided by Dr. Tanguy Seiwert (University of Chicago). Detailed characteristics of each cell line are shown in Supplementary Table 2. HN-6 cells were maintained in DMEM medium with 10% fetal bovine serum, 1% penicillin/streptomycin, and 2 nM L-glutamine. HN-SCC-151 cells were maintained in DMEM/F12 medium, 10% fetal bovine serum, 1% penicillin/streptomycin and 2 nM L-glutamine. All cells were maintained at 37°C in humid air with 5% CO2 condition. SCCHN cell lines were treated with recombinant human IFN-beta 8499-IF-010 (R&D systems) 24 hours prior to collection.

### siRNA transfection

MISSION_ siRNA oligonucleotide duplexes were purchased from Sigma–Aldrich for targeting the human SMYD3 transcripts (SASI_Hs02_00355988). siNegative control (siNC), which consists of three different oligonucleotide duplexes, were used as control siRNAs (Cosmo Bio, Tokyo, Japan). The siRNA sequences are described in Supplementary Table 3. SCCHN cells were plated overnight in 100mm culture dishes and were transfected with siRNA duplexes (50 nM final concentration) using Lipofectamine RNAimax (Thermo Fisher Scientific) for 72 hours at a confluence of ~ 50%.

### Antibodies

The following primary antibodies were used for western blotting (WB): anti-SMYD3 (D2Q4V) (rabbit #12859); Cell Signaling Technology; dilution used in WB: 1:1000, anti-TAP1 (rabbit #12341); Cell Signaling Technology; dilution used in WB: 1:1000, anti-histone H3 (tri methyl K4) (ab8580); Abcam; dilution used in WB: 1:10000, anti-histone H3 (ab1791); Abcam; dilution used in WB: 1:20000), anti-β-actin (AC-15) (mouse #A5441); Sigma-Aldrich; dilution used in WB: 1:5000.

### Western blotting

Nuclear extracts were prepared using the Nuclear Extract kit (Active Motif) to examine protein levels of SMYD3 and H3K4me3; cytoplasmic extracts were prepared using the NE-PER nuclear and cytoplasmic extraction kit (78833, ThermoFisher Scientific) to examine protein levels of TAP1. Histone H3 and β-actin were used as loading controls for the nuclear and cytoplasmic extracts, respectively. Protein extracts were separated by SDS-PAGE and blotted to nitrocellulose membrane. Nitrocellulose membrane was first incubated with each primary antibody as described in the Antibodies section, and then protein bands were detected by incubating secondary antibody: horseradish peroxidase (HRP)-conjugated antibodies (GE Healthcare, Little Chalfont, UK) and visualized with enhanced chemiluminescence (Thermo Fisher Scientific).

### Quantitative real-time PCR

Specific primers for human *GAPDH* (housekeeping gene), *CXCL9*, *CXCL10*, *CXCL11*, *TAP1*, *and SMYD3* were designed. Primer sequences are described in Supplementary Table 3. PCR reactions were performed using ViiA 7 real-time PCR system (Thermo Fisher Scientific, Waltham, MA) following the manufacture's protocol.

### ELISA assays

The protein levels of CXCL9, CXCL10 and CXCL11 were measured from cell culture supernatants using commercially available kits (Thermo Scientific) according to the manufacturer's instructions. Supernatants were stored at -20°C until respective chemokine assays were performed. Samples were run in duplicate.

### Statistical analysis

For comparison of gene expression between groups, unpaired, two-sided Student's t-test was used. Pearson product-moment correlation coefficient r was computed for measuring statistical dependence between expression of different genes. *P* < 0.05 was considered statistically significant and denoted as follows: ^*^P < 0.05, ^**^P < 0.01, ^***^P < 0.001, ^****^P < 0.0001. Statistical analysis was performed using R.

## SUPPLEMENTARY MATERIALS FIGURES AND TABLES


