# Supplementary material for: Protein methyltransferases and demethylases dictate CD8+ T-cell exclusion in squamous cell carcinoma of the head and neck
Source: Oncotarget. 2017 Nov 22;8(68):112797–808. doi: 10.18632/oncotarget.22627 (PMC5762551; doi:10.18632/oncotarget.22627)
Supplement: Supplementary file 1 [file oncotarget-08-112797-s001.pdf]

Protein methyltransferases and demethylases dictate CD8+ T-cell exclusion in squamous cell carcinoma of the head and neck

SUPPLEMENTARY MATERIALS

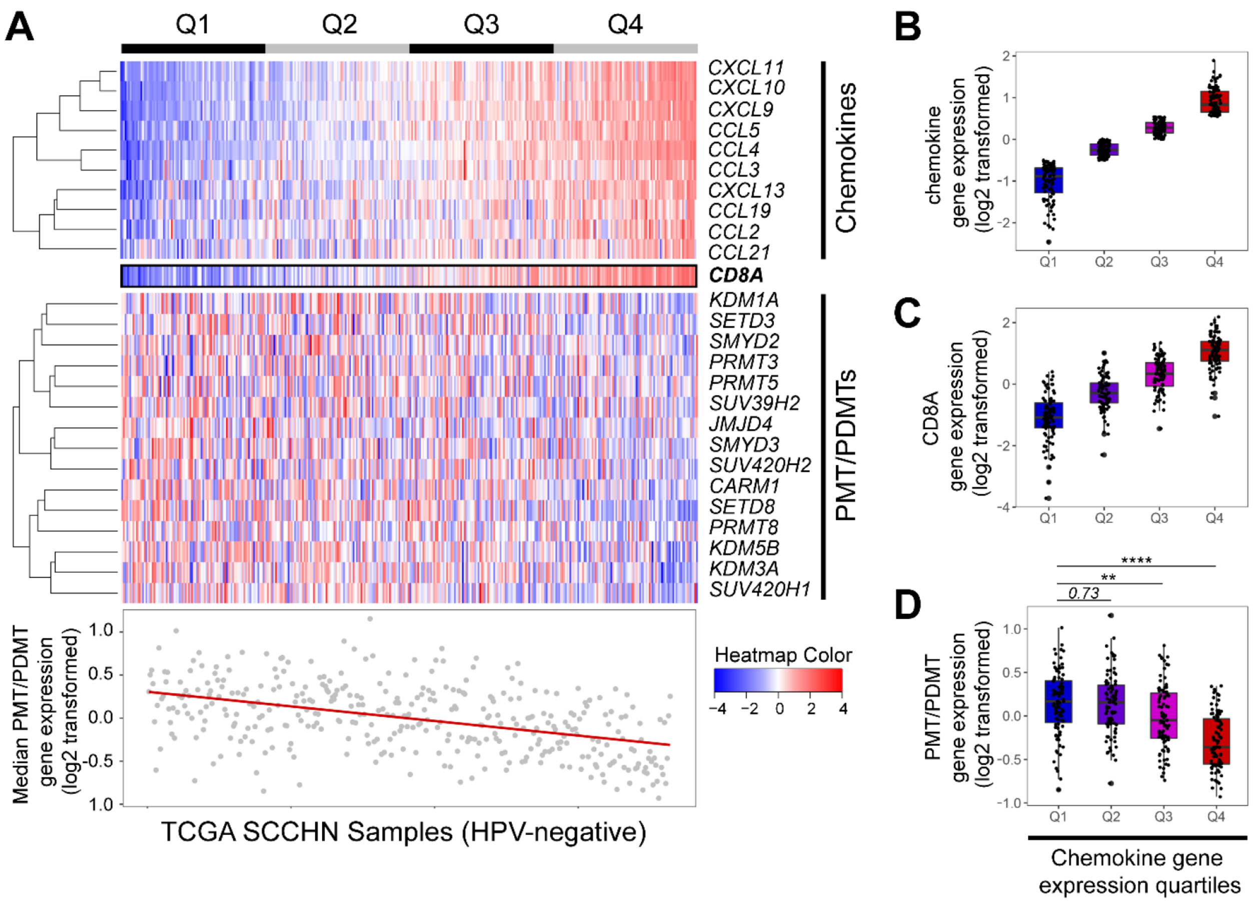

Supplementary Figure 1: Heatmap of 15 candidate PMTs/PDMTs with chemokine signature in the TCGA database for HPV-negative squamous cell carcinoma of the head and neck (SCCHN; HPV-negative $n = 364$) (chemokine expression quartiles Q1 to Q4 shown). Heatmaps were generated using the normalized and log2-transformed RNA-seq gene expression data. **(A)**, top: Expression heatmap of the 10 chemokine genes (left to right: samples were sorted by median chemokine gene expression, lower to higher), and CD8A gene expression. (A), middle: Expression heatmap of the 15 PMT/PDMT genes (same sample order as the chemokine gene heatmap). (A), bottom: Scatter plot showing the trend of PMT/PDMT gene expression as chemokine gene expression increases. Each dot represents one sample. Median expression of the 15 PMT/PDMT genes is shown on the y-axis. Red line shows the smoothened linear regression of the data plots (sample order same as per the chemokine gene heatmap). **(B-D)** Box plot showing the expression of (B) chemokines, (C) CD8A and (D) PMT/PDMT genes in samples grouped by chemokine expression quartiles Q1 to Q4. $P < 0.05$ was considered statistically significant, and significance is indicated by: * $P < 0.05$, ** $P < 0.01$, *** $P < 0.001$, **** $P < 0.0001$.

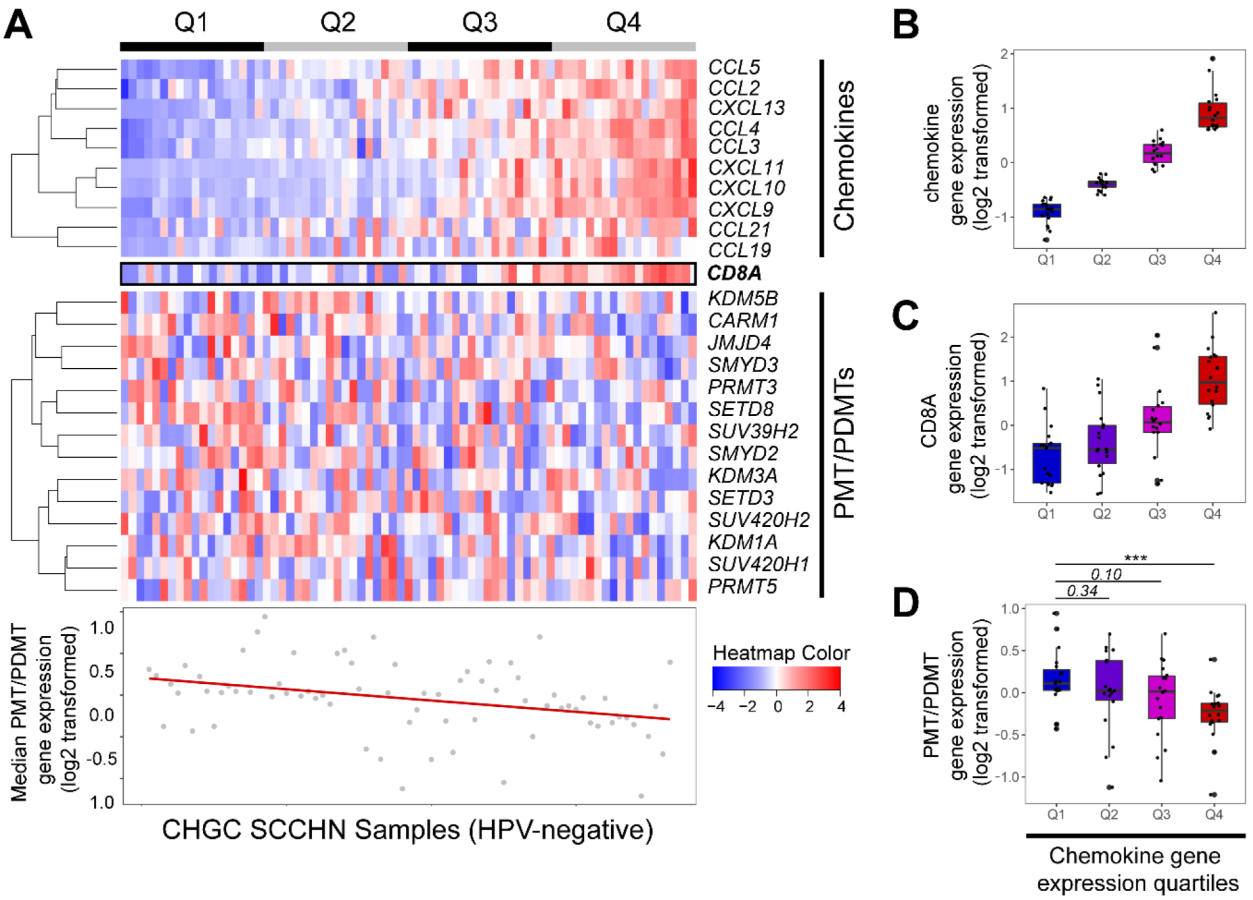

Supplementary Figure 2: Heatmap of 15 candidate PMTs/PDMTs with chemokine signature in the CHGC database for HPV-negative squamous cell carcinoma of the head and neck (SCCHN; HPV-negative n = 73) (chemokine expression quartiles Q1 to Q4 shown). Heatmaps were generated using the normalized and log2-transformed RNA-seq gene expression data. **(A)**, top: Expression heatmap of the 10 chemokine genes (left to right: samples were sorted by median chemokine gene expression, lower to higher), and CD8A gene expression. (A), middle: Expression heatmap of the 15 PMT/PDMT genes (same sample order as the chemokine gene heatmap). (A), bottom: Scatter plot showing the trend of PMT/PDMT gene expression as chemokine gene expression increases. Each dot represents one sample. Median expression of the 15 PMT/PDMT genes is shown on the y-axis. Red line shows the smoothed linear regression of the data plots (sample order same as per the chemokine gene heatmap). **(B-D)** Box plot showing the expression of (B) chemokines, (C) CD8A and (D) PMT/PDMT genes in samples grouped by chemokine expression quartiles Q1 to Q4. $P < 0.05$ was considered statistically significant, and significance is indicated by: * $P < 0.05$, ** $P < 0.01$, *** $P < 0.001$, **** $P < 0.0001$.

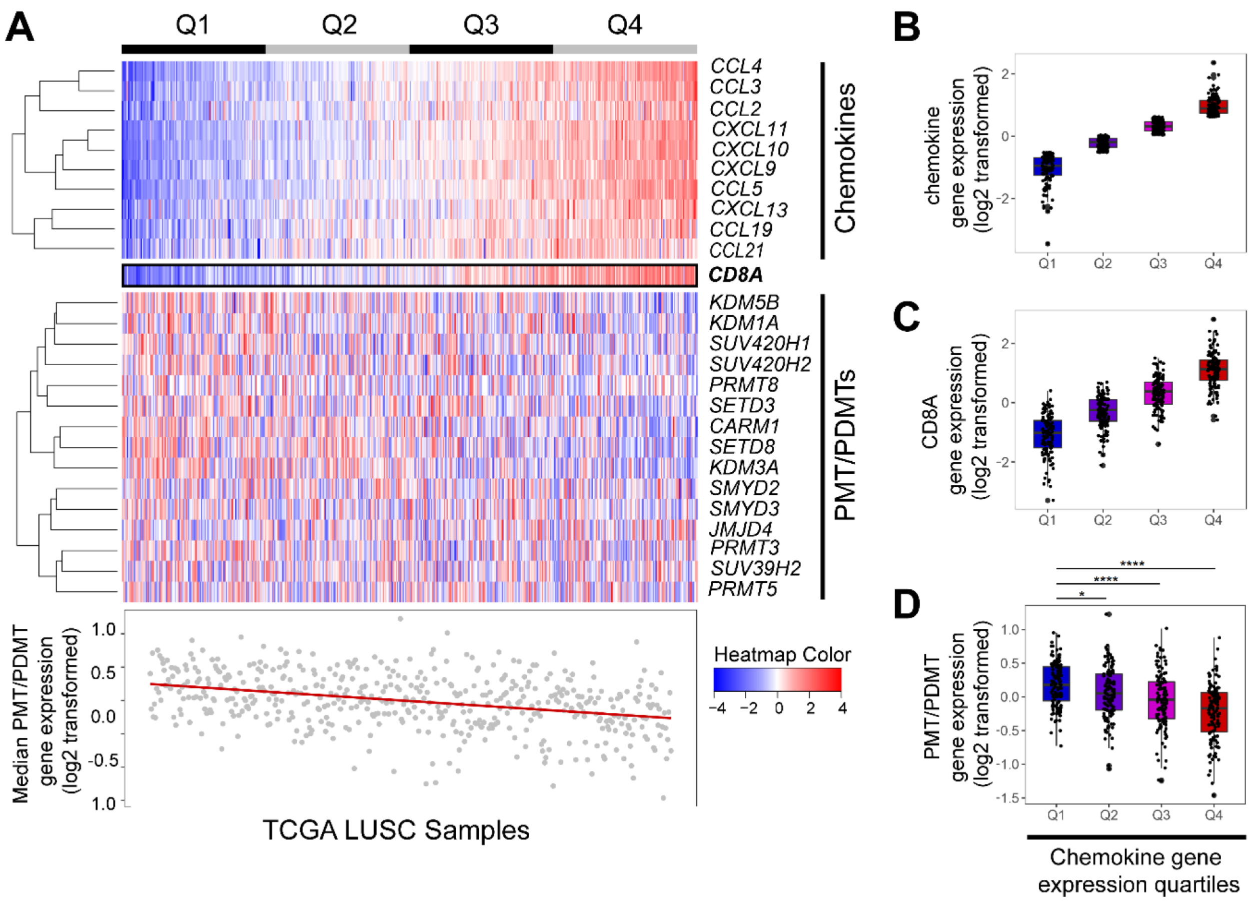

Supplementary Figure 3: Heatmap of 15 candidate PMTs/PDMTs with chemokine signature in the TCGA database for lung squamous cell carcinoma (LUSC; *n* = 488) (chemokine expression quartiles Q1 to Q4 shown). Heatmaps were generated using the normalized and log2-transformed RNA-seq gene expression data. **(A)**, top: Expression heatmap of the 10 chemokine genes (left to right: samples were sorted by median chemokine gene expression, lower to higher), and CD8A gene expression. (A), middle: Expression heatmap of the 15 PMT/PDMT genes (same sample order as the chemokine gene heatmap). (A), bottom: Scatter plot showing the trend of PMT/PDMT gene expression as chemokine gene expression increases. Each dot represents one sample. Median expression of the 15 PMT/PDMT genes is shown on the y-axis. Red line shows the smoothed linear regression of the data plots (sample order same as per the chemokine gene heatmap). **(B-D)** Box plot showing the expression of (B) chemokines, (C) CD8A and (D) PMT/PDMT genes in samples grouped by chemokine expression quartiles Q1 to Q4. $P < 0.05$ was considered statistically significant, and significance is indicated by: * $P < 0.05$, ** $P < 0.01$, *** $P < 0.001$, **** $P < 0.0001$.

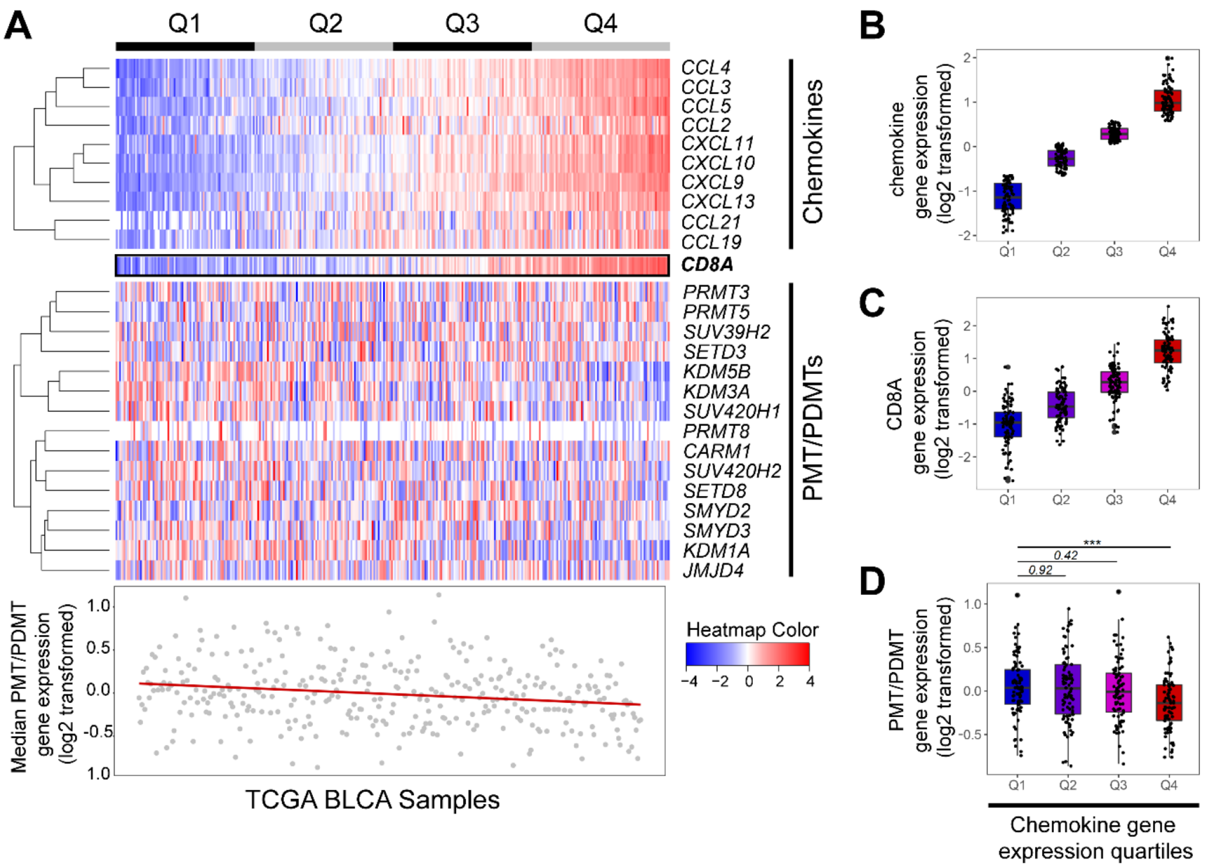

AQ 4 **Supplementary Figure 4: Heatmap of 15 candidate PMTs/PDMTs with chemokine signature in the TCGA database for bladder urothelial carcinoma (BLCA; *n* = 344) (chemokine expression quartiles Q1 to Q4 shown).** Heatmaps were generated using the normalized and log2-transformed RNA-seq gene expression data. **(A)**, top: Expression heatmap of the 10 chemokine genes (left to right: samples were sorted by median chemokine gene expression, lower to higher), and CD8A gene expression. (A), middle: Expression heatmap of the 15 PMT/PDMT genes (same sample order as the chemokine gene heatmap). (A), bottom: Scatter plot showing the trend of PMT/PDMT gene expression as chemokine gene expression increases. Each dot represents one sample. Median expression of the 15 PMT/PDMT genes is shown on the y-axis. Red line shows the smoothed linear regression of the data plots (sample order same as per the chemokine gene heatmap). **(B-D)** Box plot showing the expression of (B) chemokines, (C) CD8A and (D) PMT/PDMT genes in samples grouped by chemokine expression quartiles Q1 to Q4. $P < 0.05$ was considered statistically significant, and significance is indicated by: * $P < 0.05$, ** $P < 0.01$, *** $P < 0.001$, **** $P < 0.0001$.

Supplementary Table 1: List of 53 protein methyltransferase (PMT) and protein demethylase (PDMT) genes interrogated for negative correlations with chemokines and antigen presenting machinery (APM) components

PMTs	PDMTs	Chemokines	APMs and HLA class I
NSD1	KDM1A	CCL2	B2M
NSD2	KDM2A	CCL3	TAP1
NSD3	KDM2B	CCL4	TPA2
MLL	KDM3A	CCL5	TAPBP
MLL2	KDM3B	CXCL9	CALR
MLL3	JMJD1C	CXCL10	CANX
MLL4	KDM4A	CXCL11	HLA-A
MLL5	KDM4B	CXCL13	HLA-B
PRDM14	KDM4C	CCL19	HLA-C
SMYD2	KDM4D	CCL21	
SMYD3	KDM5A		
SETMAP	KDM5B		
SETD1A	KDM5C		
SETD3	KDM5D		
SETD7	KDM6A		
PRMT1	KDM6B		
PRMT2	JMJD4		
PRMT3	JMJD5		
PRMT5			
PRMT6			
PRMT7			
PRMT8			
PRMT10			
SUV420H1			
SUV420H2			
SUV39H1			
SUV39H2			
EHMT1			
EHMT2			
SETDB1			
SETDB2			
SETD8			
EZH2			
KDM5B			
KDM5C			

Supplementary Table 2: Characteristics of SCCHN cell lines

Cell name	TNM stage	Specimen site	Gender	HPV status
HN-6	T2N0	Oral cavity	Male	Negative
HN-SCC-151	T3N0	Tongue	-	Negative

Supplementary Table 3: Primer sequences for *GAPDH*, *CXCL9*, *CXCL10*, *CXCL11*, *TAP1*, *SMYD3* and siRNA sequences for siNC and siSMYD3

Gene Name	Primer Sequence
GAPDH-f	5' CGACCACTTTGTCAAGCTCA 3'
GAPDH-r	5' GGTTGAGCACAGGGTACTTTATT 3'
CXCL9-f	5' GTGGTGTTCTTTTCCTCTTGGG 3'
CXCL9-r	5' ACAGCGACCCTTTCTCACTAC 3'
CXCL10-f	5' CTCCAGTCTCAGCACCATGA 3'
CXCL10-r	5' GCTCCCCTCTGGTTTTAAGG 3'
CXCL11-f	5' GTTCAAGGCTTCCCCATGTTC 3'
CXCL11-r	5' ATAAGCCTTGCTTGCTTCGATTTG 3'
TAP1-f	5' CTGTGGCACAAACTCGGGAG 3'
TAP1-r	5' ATCTCCCCAAGAGAGGAGAGG 3'
SMYD3-f	5' GTTCGATTGTGTTCAATGGGCCCCAC 3'
SMYD3-r	5' TCCTCACTGGTCATCAGCATATC 3'

siRNA Name		Sequence
siNegative Control (siNC)	Target#1	Sense: 5' AUCCGCGCGAUAGUACGUA 3'<br>Antisense: 5' UACGUACUAUCGCGCGGAU 3'
	Target#2	Sense: 5' UUACGCGUAGCGUAAUACG 3'<br>Antisense: 5' CGUAUUACGCUACGCGUAA 3'
	Target#3	Sense: 5' UAUUCGCGCGUAUAGCGGU 3'<br>Antisense: 5' ACCGCUAUACGCGCGAAUA 3'
siSMYD3		Sense: 5' GCAAGUAUGGAAGGAAGUU 3'<br>Antisense: 5' AACUUCCUUCCAUACUUGC 3'

Supplementary Table 4: PMT/PDMTs that correlate negatively ($P < 0.05$, unadjusted) with chemokines or HLA class I and APM molecules in squamous cell carcinoma of the head and neck, lung squamous, esophageal squamous carcinoma cell lines ($n = 184$) of the CCLE RNA sequencing expression database*

PMT/PDMTs	Chemokines	HLA class I and APM molecules
**PMTs**		
**SMYD2**	**CCL5**	**B2M, HLA-A/B, IRF1, TAP1, TAPBP**
**SMYD3**	CXCL10, CXCL11, CCL5	TAP1, TAP2, TAPBP, HLA-B/C, B2M**, IRF1**
**SUV39H2**	CXCL10, CXCL11, CCL5	B2M, HLA-A/B/C, IRF1, TAP1, TAP2, **CALR, CANX, TAPBP**
**SUV420H1**		B2M, HLA-B, TAP1, TAP2, **CALR, CANX**
**SUV420H2**	CCL2, **CCL5**	**B2M, CALR, CANX, HLA-A/B/C, IRF1, TAP1, TAP2, TAPBP**
**SETD3**	**CXCL10**	
**SETD8**	**CXCL11, CCL5**	CALR, HLA-B, TAP1, **IRF1, TAP1, TAPBP**
**PRMT3**		TAPBP, CALR, **HLA-A/B/C, IRF1,**
**PRMT5**		TAPBP, **B2M, HLA-C, IRF1**
**PRMT8**		**TAP1, TAP2**
**PDMTs**		
**KDM1A**	**CCL5**	**B2M, CALR, CANX, HLA-A/B/C, IRF1, TAP1, TAP2, TAPBP**
**KDM3A**		HLA-B/C, TAP1, TAP2, CALR, B2M, **CANX**
**KDM5B**		**B2M, CANX, HLA-A/B, TAP1, TAP2**
**JMJD4**		**B2M**

Abbreviations: PMT: protein methyltransferase, PDMT: protein demethylase, APM: antigen presenting machinery, CCLE: Cancer Cell Line Encyclopedia.
*Only genes expressed in at least 50 samples were used for Pearson's correlation analysis.
**Regular font represents chemokines or HLA class I and APM components that negatively correlated with the candidate PMT/PDMTs both in the TCGA SCCHN database as well as the CCLE database. Bold font represents chemokines or HLA class I and APM components that negatively correlated with candidate PMT/PDMTs only in the CCLE database.
